# High-timing-precision detection of single X-ray photons by superconducting nanowires

**DOI:** 10.1093/nsr/nwad102

**Published:** 2023-04-18

**Authors:** Shuya Guo, Jingrou Tan, Hengbin Zhang, Jinguang Wang, Tianhao Ji, Labao Zhang, Xiaolong Hu, Jian Chen, Jun Xie, Kai Zou, Yun Meng, Xiaomin Bei, Ling-An Wu, Qi Chen, Hao Wang, Xuecou Tu, Xiaoqing Jia, Qing-Yuan Zhao, Lin Kang, Peiheng Wu

**Affiliations:** Research Institute of Superconductor Electronics, Nanjing University, Nanjing 210093, China; Research Institute of Superconductor Electronics, Nanjing University, Nanjing 210093, China; Qian Xuesen Laboratory of Space Technology, Beijing 100094, China; Beijing National Laboratory of Condensed Matter Physics, Institute of Physics, Chinese Academy of Sciences, Beijing 100190, China; Research Institute of Superconductor Electronics, Nanjing University, Nanjing 210093, China; Research Institute of Superconductor Electronics, Nanjing University, Nanjing 210093, China; Hefei National Laboratory, Hefei 230088, China; School of Precision Instrument and Optoelectronic Engineering, Tianjin University, Tianjin 300072, China; Key Laboratory of Optoelectronic Information Science and Technology, Ministry of Education, Tianjin 300072, China; Research Institute of Superconductor Electronics, Nanjing University, Nanjing 210093, China; Qian Xuesen Laboratory of Space Technology, Beijing 100094, China; School of Precision Instrument and Optoelectronic Engineering, Tianjin University, Tianjin 300072, China; Key Laboratory of Optoelectronic Information Science and Technology, Ministry of Education, Tianjin 300072, China; School of Precision Instrument and Optoelectronic Engineering, Tianjin University, Tianjin 300072, China; Key Laboratory of Optoelectronic Information Science and Technology, Ministry of Education, Tianjin 300072, China; Qian Xuesen Laboratory of Space Technology, Beijing 100094, China; Beijing National Laboratory of Condensed Matter Physics, Institute of Physics, Chinese Academy of Sciences, Beijing 100190, China; Research Institute of Superconductor Electronics, Nanjing University, Nanjing 210093, China; Research Institute of Superconductor Electronics, Nanjing University, Nanjing 210093, China; Research Institute of Superconductor Electronics, Nanjing University, Nanjing 210093, China; Research Institute of Superconductor Electronics, Nanjing University, Nanjing 210093, China; Hefei National Laboratory, Hefei 230088, China; Research Institute of Superconductor Electronics, Nanjing University, Nanjing 210093, China; Research Institute of Superconductor Electronics, Nanjing University, Nanjing 210093, China; Hefei National Laboratory, Hefei 230088, China; Research Institute of Superconductor Electronics, Nanjing University, Nanjing 210093, China; Hefei National Laboratory, Hefei 230088, China

**Keywords:** single-photon detection, timing precision, superconductor, ultrafast phenomenon, pulsar X-ray navigation

## Abstract

Precisely acquiring the timing information of individual X-ray photons is important in both fundamental research and practical applications. The timing precision of commonly used X-ray single-photon detectors remains in the range of one hundred picoseconds to microseconds. In this work, we report on high-timing-precision detection of single X-ray photons through the fast transition to the normal state from the superconductive state of superconducting nanowires. We successfully demonstrate a free-running X-ray single-photon detector with a timing resolution of 20.1 ps made of 100-nm-thick niobium nitride film with an active area of 50 *μ*m by 50 *μ*m. By using a repeated differential timing measurement on two adjacent X-ray single-photon detectors, we demonstrate a precision of 0.87 ps in the arrival-time difference of X-ray photon measurements. Therefore, our work significantly enhances the timing precision in X-ray photon counting, opening a new niche for ultrafast X-ray photonics and many associated applications.

## Teaser

The research reports a free-running X-ray single-photon detector with a timing resolution of 20 ps and demonstrates a precision of 0.87 ps in the arrival-time difference of X-ray photon measurements.

## INTRODUCTION

Since Röntgen discovered X-ray radiation and recorded the first X-ray image of his wife’s hand and ring [1], X-ray radiation has played an important role in science and technology, for example, in astronomy to explore outer space [2], in medicine to diagnose diseases [3] and in crystallographics to study solids [4,5]. Among these applications, acquiring the timing information that the X-ray carries is critical to delivering high-precision timing information for X-ray pulsar-based navigation [6–8], to recording ultrafast X-ray photography [9] and to revealing the ultrafast dynamics of individual electrons or nuclei [10–12]. Although the detection of X-ray radiation can be as fast as the order of femtoseconds or even attoseconds [13], the timing precision of the direct detection of individual X-ray photons remains in the range of one hundred picoseconds to microseconds with currently available detectors, including photomultiplier tubes [14], semiconductor detectors [15], scintillator X-ray photodetectors [16], transition-edge sensors [17], kinetic inductance detectors [18] and superconducting-tunnel-junction detectors [19]. Some of them [15–18] are energy-resolving detectors.

Superconducting nanowire single-photon detectors (SNSPDs) [20] have been becoming ideal detectors of choice in many photon-counting applications in the visible, near and middle infrared (IR) spectral ranges that do not require intrinsic energy resolution. In recent years, SNSPDs have been used in quantum-key distribution [21], lunar laser ranging [22], biomedical imaging [23] and deep-space optical communication [24]. Compared with most SNSPD-optimized near-IR telecommunication wavelengths of 1550 nm [25–29], SNSPDs that target photon counting in the soft X-ray band with photon energy of several kiloelectronvolts are just emerging and are more technologically challenging [30–35]. Several materials including niobium (Nb) [30], tantalum nitride (TaN) [31,33] and tungsten silicide (W_0.8_Si_0.2_) [32], thin films (<10 nm) [34,35] and thicker films (∼100 nm) [30–33], nanowires [30–32,34,35] and microwires [33] have been explored for making X-ray SNSPDs. Thin films used in near-IR SNPSDs have low absorption coefficients of the X-ray photons due to the greater X-ray photon penetration, compared with near-IR counterparts; therefore, thicker films are required for effective absorption. However, SNSPDs made of thicker superconducting films are more likely to suffer from latching [30,36], an effect that hinders the detector from free running. In addition, SNSPDs working in the near-IR and visible spectral ranges have been extensively investigated in terms of their timing performances, including possible mechanisms inducing timing jitter [25–29,37–39], devices with ultralow timing jitter [40] as well as practical devices and systems combining high detection efficiency with high timing resolution [41,42]. In direct, dramatic contrast, the timing properties of X-ray SNSPDs remain almost completely unexplored. Therefore, it is unknown whether X-ray SNSPDs could provide exceptional timing precision on the picosecond time scale, as has been demonstrated on their visible counterparts [40]. It is also unknown what and how extrinsic and intrinsic factors affect the timing precision of X-ray SNSPDs. Finally, it is unclear how the envelope of numerous engineering efforts in device design and nanofabrication, system implementation and optimization, and testing and measurement techniques could help improve the timing precision so that, possibly, the ultimate limit of the timing precision of X-ray SNSPDs could be probed and reached.

Here we report on the first experimental investigation and analysis of the timing properties of X-ray SNSPDs. With combined optimizations in nanofabrication of devices, bias and readout circuitry, and testing and measurement facilities, we successfully demonstrate an X-ray SNSPD made of a 100-nm-thick niobium nitride (NbN) film with an active area of $50\times 50\, \mu{\rm m}^2$. It reaches an unprecedented timing precision of 20.1 ps, operating in the free-running mode that continuously detects X-ray photons without latching. We also study the dependence of the timing jitter on the geometries of the detectors, bias conditions and energy of X-ray photons, with further analysis of the extrinsic and intrinsic components of the measured total timing jitter. In particular, we perform a repeated, differential timing measurement on two adjacent X-ray SNSPDs fabricated on the same chip, and demonstrate an exceptional timing precision of 0.87 ps in measuring the arrival time of X-ray photons. Our results push the timing precision of X-ray photon counters to the picosecond regime, and, therefore, we believe that this work not only impacts the research and development of ultrafast X-ray photonics [4,5,9–12], but also finds numerous applications in X-ray pulsar navigation [7,8] and X-ray astronomy [2].

## RESULTS

**Figure 1.**
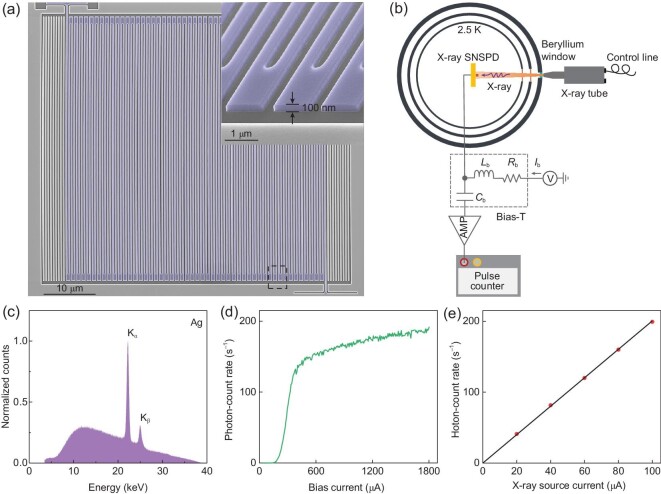
Characterization of the superconducting nanowire single X-ray photon detector (X-ray SNSPD) using a continuous X-ray source. (a) False-colored scanning-electron micrograph of an example X-ray SNSPD. The thickness of the NbN film is 100 nm, the width of the nanowire is 250 nm, the fill factor is 0.5 and the active area is $50\times 50\, \mu{\rm m}^2$. Inset: a tilted, enlarged view of the false-color scanning-electron micrograph of the nanowire. (b) Experimental setup with a continuous X-ray source for characterizing the photon-count rate (PCR). AMP: amplifier. (c) The energy spectrum of the continuous X-ray sources, emitted from the silver (Ag) target, was measured by a mercury cadmium telluride detector in photon-counting mode. (d) PCR versus bias current. (e) PCR of the X-ray SNSPD at $I_{\rm b}=1760\, \mu$A versus the current of the X-ray source. The black line is a linear fitting to the measured data shown with red dots.

The false-colored scanning-electron micrograph (SEM) of a typical X-ray SNSPD we fabricated (see the Methods section for the nanofabrication process) is shown in Fig. 1(a), demonstrating the good uniformity of nanowires. In order to optimize its timing precision, several crucial factors in the process of designing and making the detectors were taken into account. Polycrystalline NbN is the material of choice because compared with amorphous superconducting materials (e.g. W_0.8_Si_0.2_ [32]), the higher density of the supercurrent in a NbN nanowire could decrease the noise- and inhomogeneity-induced timing jitter [25–29]. All detectors were fabricated with 100-nm-thick films (see the inset of Fig. 1(a)) because the thicker films, compared with the sub-10-nm films [34,35], permit better X-ray absorption (see Fig. 1 within the online supplementary material). Another advantage of thicker films is better uniformity, reducing the inhomogeneity-induced timing jitter [27,29]. The relatively low kinetic inductance also helps reduce the timing jitter [26,27]. All the turns of the meandering structure were optimized in their design to mitigate the current-crowding effect [43], to increase the switching current of the nanowire, and to decrease the timing jitter. We chose an active area of $50\times 50\, \mu{\rm m}^2$ (Fig. 1(a)) as the major device under study, which took into account a large enough receiving aperture for the X-ray photons and the fact that an even larger area would deteriorate the timing resolution of the resulting detector. The envelope of all these engineering factors and optimizations is the key to achieving X-ray SNSPDs with unprecedented timing resolution and precision.

**Figure 2.**
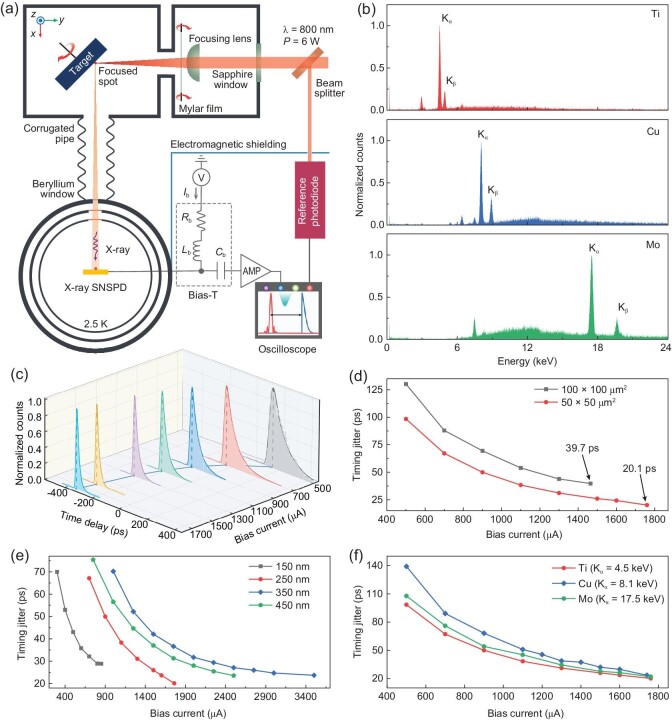
Timing properties of the X-ray SNSPD. (a) Schematic of the experimental setup. Light from a femtosecond laser, with a central wavelength of 800 nm and peak power of 6 W, was focused on the rotating target to generate the femtosecond X-ray pulses [44]. An X-ray illuminated the X-ray SNSPD, the temporal traces of the output signals were recorded and the timing jitter of the X-ray SNSPD was measured. Electromagnetic shielding was used to avoid interference with the measurement equipment in the ambient environment. (b) The energy spectra of the femtosecond X-ray photons generated from the titanium (Ti), copper (Cu) and molybdenum (Mo) targets were measured by a mercury cadmium telluride detector in photon-counting mode. (c) Histograms of the time delays measured as functions of the bias current of the X-ray SNSPD with a $50\times 50\, \mu{\rm m}^2$ active area and a 250-nm-wide nanowire, illuminated by the X-ray photons generated from the Ti target. (d) Timing jitter as a function of the bias current for a $50\times 50\,\mu{\rm m}^2$ detector and a $100\times 100\, \mu{\rm m}^2$ detector, both with 250-nm nanowire width. The Ti target was used. (e) Timing jitter as a function of bias current for 150-, 250-, 350- and 450-nm-wide nanowires, all with a $50\times 50\, \mu{\rm m}^2$ active area. The Ti target was used. (f) Timing jitter as a function of the bias current for the $50\times 50\, \mu{\rm m}^2$ X-ray SNSPD, measured with X-ray photons generated from three types of target, Ti, Cu and Mo.

We employed a continuous X-ray source to confirm that the detector was working in the free-running, single-photon-detection regime and to measure its photon-count rate (PCR) as a function of the bias current. Figure 1(b) presents the experimental setup. If the commonly used current-biased sources were used to bias the detector [25,26], the detector would suffer from latching, meaning that it could not continuously detect X-ray photons in the free-running mode [30]. Latching occurred because of the low kinetic inductance and the resulting too fast current dynamics and because of the thicker films and the resulting slow dissipation of the absorbed energy. Therefore, we used a homemade voltage-bias circuitry (see the Methods section for details) to avoid latching. The resistance, inductance and capacitance of *R*_b_, *L*_b_ and *C*_b_ are 5 Ω, 500 nH and 10 pF, respectively. The reduced resistance adapts to the lower hotspot resistance due to the thicker NbN film. The flux of the X-ray photons emitted from the X-ray tube was controlled by the current and voltage in the tube, and it increased linearly with the current at a fixed voltage. The X-ray SNSPD was installed on the cold head with a base temperature of 2.5 K of a pulse-tube cryostat. The switching current, *I*_sw_, of the X-ray SNSPD was measured to be 1800 *μ*A at this temperature. Without X-ray illumination, we could not measure any counts within 10^3^ s at the bias current close to *I*_sw_, meaning that the dark-count rate was negligibly low and <10^−3^ counts per second (cps). The X-ray photons illuminated the detector through a beryllium window of the cryostat and two polyimide windows on the two stages of the thermal shields. The target of the X-ray tube was silver, and its energy spectrum is presented in Fig. 1(c), featuring a relatively broad energy distribution with a sharp ${\rm K_{\rm {{\alpha }}}}$ peak at 22.2 keV and a sharp K_β_ peak at 24.9 keV. Figure 1(d) presents PCR as a function of the bias current, *I*_b_, of the X-ray SNSPD, fixing the bias current and accelerating the voltage of the X-ray tube to 100 *μ*A and 40 kV, respectively. One important feature of this curve is the low threshold bias current, 207 *μ*A, above which PCR becomes prominent. Another feature is that, when the bias current is larger than 385 *μ*A, the increase in PCR with the bias current slows down, showing a trend of saturation. We attempted to fit this curve with two segments of linear functions and presented the fitting in Fig. 2 within the online supplementary material. For X-ray photons with an energy of 10 keV, the photoelectric effect is the dominant effect in their interaction with NbN. According to the database [45], the absorbance due to Compton scattering is 35 times less than that due to the photoelectric effect. On the other hand, researchers reported that the absorbed energy could generate hot phonons as secondary particles to excite nanowires made of thin films [34], and we speculate that a similar effect may exist in our devices, which may explain the increase in PCR as a function of bias current in the saturated detection efficiency region. Figure 1(e) presents PCR as a function of the bias current on the X-ray tube whose accelerating voltage was fixed at 40 kV. The bias current, *I*_b_, was 1760 *μ*A. The good linear fitting confirmed that the detector was working in the single-photon-detection regime.

**Figure 3.**
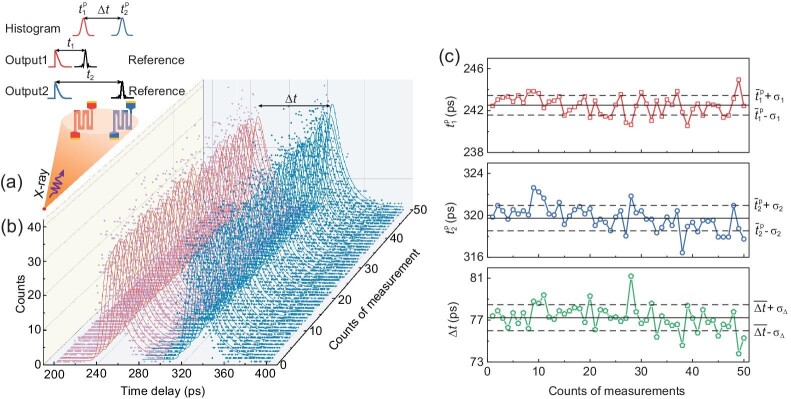
Repeated differential timing measurements. (a) Schematics showing the principle of the differential timing measurements. Two adjacent X-ray SNSPDs were employed to cancel some timing jitter. (b) Histograms of the time delays, measured by the two X-ray SNSPDs. For each X-ray SNSPD, each histogram contained 800 time delays and 50 histograms were measured and recorded. Here Δ*t* is the time difference between the two exponentially modified Gaussian peaks in each group. (c) Timing for each individual X-ray SNSPD (red and blue) and the time difference between the two (green). The solid black lines show the mean values of timing and the dashed black lines show the standard deviations.

To investigate the timing properties of X-ray SNSPDs, we used a home-built setup as presented in Fig. 2(a) and detailed in the Methods section to measure their timing jitter. Accordingly, Fig. 3 within the online supplementary material shows a photograph of the experimental setup with its major components labeled. In this setup, the ultrafast laser illuminated the target in the vacuum chamber and generated femtosecond X-ray pulses. The average number of X-ray photons in each pulse was estimated to be 1.8 × 10^8^ so that the probability that each pulse contains more than one X-ray photon arriving at the active area of the X-ray SNSPD was only approximately $3\%$ (see Fig. 4 and Note 1 within the online supplementary material for details). We used three types of target, titanium (Ti), copper (Cu) and molybdenum (Mo), whose energy spectra were measured and are presented in Fig. 2(b).

We studied the dependence of timing jitter on the bias current and the geometries of the nanowires, and on the types of target. Figure 2(c) presents the histograms of the time delays measured as a function of the bias current. In this study, the X-ray SNSPD had an active area of $50\times 50\, \mu{\rm m}^2$, and the target used for generating the X-ray photons was Ti. Considering the geometry of the experimental setup (Fig. 4 within the online supplementary material), we estimated that the rate of X-ray photons coupled to the active area of the X-ray SNSPD was 260 cps. Because the PCR from the X-ray SNSPD was approximately 10 cps at a bias current of 1760 *μ*A (therefore, the device efficiency, excluding coupling efficiency, was estimated to be $10 / 260 = 3.8\%$), much lower than the repetition rate of the reference signal, 1 kHz, we used the output signal from the X-ray SNSPD as the trigger and measured the time delay of the reference signal relative to the output from the X-ray SNSPD using a 33-GHz real-time oscilloscope. From the PCR and the estimated rate of X-ray photons coupled to the active area, we estimated the device efficiency, excluding coupling efficiency, to be $10 / 260 = 3.8\%$, which should be compared with the calculated absorptance, 2.4%, of the detector with a fill factor of 50%. The estimated device efficiency was slightly larger than the calculated absorptance, and we attributed this discrepancy to error in the estimation of the flux of X-ray photons. Figure 5 within the online supplementary material shows the temporal trace of the output pulse without any ratio frequency (RF) amplifiers. An exponential-decay fitting of the falling edge shows a time constant of 8.9 ns. In addition to the exponential decay, the pulse contains a long tail on a time scale of a few microseconds. We attributed this long tail to the impedance mismatch. In Fig. 2(c) and throughout this paper, each histogram of the measured time delays was normalized to its maximum and was fitted by an exponentially modified Gaussian function (see Fig. 6 and Note 2 within the online supplementary material for details) [46], whose full width at half maxima (FWHM) was used to quantify the timing jitter. At low bias current, the histograms show more prominent tails at the large time delay and larger timing jitter, while at high bias current, the histograms are more Gaussian-like and the timing jitter decreases (see Figs 7 and 8, and Note 3 within the online supplementary material for details).

Figure 2(d) summarizes the measured timing jitter, as a function of the bias current, of this X-ray SNSPD with a $50\times 50\, \mu{\rm m}^2$ active area and presents a comparison with another X-ray SNSPD with a large area, $100\times 100\, \mu{\rm m}^2$. The former exhibited a larger switching current, 1800 *μ*A, and the lowest timing jitter was 20.1 ps; in comparison, the latter exhibited a relatively lower switching current, 1600 *μ*A, and the lowest timing jitter was 39.7 ps. These results indicate that the X-ray SNSPDs with larger areas probably contain more localized defects and spatially distributed inhomogenies that limit the switching current and deteriorate the timing resolution and have higher geometric timing jitter due to the transmission-line effect of the nanowire [37]. These trends were strikingly (considering the fact that we used the 100-nm-thick film here) similar to those of regular SNSPDs made of sub-10-nm-thick films. However, the minimal timing jitter, as low as 20.1 ps, would be impossible for regular SNSPDs made of thin films that cover the same active area of $50\times 50\, \mu{\rm m}^2$. Figure 2(e) presents the timing jitter as a function of bias current for 150-, 250-, 350- and 450-nm-wide nanowires covering the same active area of $50\times 50\, \mu{\rm m}^2$. For even smaller devices, we only measured the timing jitter with the highest bias currents because the PCR was too low and the measurements of the histograms at a lower bias current took an unacceptably long time. Table 1 summarizes the geometric dependence of the timing jitter. For very small detectors, timing jitter as low as 15 ps was measured, but, of course, at the expense of overall system detection efficiency and PCR. We also measured the dependence of the timing jitter on the energy of the X-ray photons. Figure 2(e) presents this dependence for three types of target, titanium (Ti), copper (Cu) and molybdenum (Mo), for the major X-ray SNSPD under study. The results lead us to conclude that over a wide range of energies of X-ray photons, the X-ray SNSPD can achieve timing jitter as low as ≈20 ps.

**Table 1.** Geometry, switching current and the minimal timing jitter for X-ray SNSPDs.

		Total length	Switching	Minimal timing
Detectors		(*μ*m)	current (*μ*A)	jitter (ps)
Various areas with	$10\times 10\, \mu{\rm m}^2$	200	2012	15.0
250-nm-wide	$20\times 20\, \mu{\rm m}^2$	800	1962	17.9
nanowires	$50\times 50\, \mu{\rm m}^2$	5000	1800	20.1
	$100\times 100\, \mu{\rm m}^2$	20 000	1600	39.7
Various widths with	150 nm	8333	900	28.5
a $50\times 50\, \mu{\rm m}^2$ area	250 nm	5000	1800	21.9
	350 nm	3571	2500	25.2
	450 nm	2778	3500	24.2

We attempted to analyze the origin of the timing jitter of X-ray SNSPDs and inferred their intrinsic timing jitter. Note that, although the mechanisms of single X-ray photon detection using SNSPDs have previously been outlined [30,34], a quantitative model is still absent. In what follows, we use the framework for analyzing the timing jitter of regular SNSPDs working in the visible and near-IR regions that has been established [25–29,37–39], and we here state this assumption explicitly. In Figs. 9 and 10 within the online supplementary material we measured and calculated the timing jitters induced by electronic noise, the fast photodetector, the femtosecond laser and the transmission-line effect in detail to be 3.7, 0.98, 0.1 and 3.5 ps, respectively, for the major X-ray SNSPD under study, biased at 1760 *μ*A. Note that the geometric timing jitter due to the transmission-line effect was measured on another X-ray SNSPD with the identical design but with the dual-port bias and readout circuitry [37]. See Supplementary Fig. 10 for more details. Additionally, the vibration of the target resulted in the fluctuation of the length of the optical path. The peak-to-peak vibration was 300 *μ*m and, therefore, we estimated that the timing jitter induced by this effect was <0.5 ps. By quadratically subtracting these components from the measured total timing jitter of 20.1 ps, we infer that the intrinsic timing jitter, probably including the Fano-fluctuation-induced [28], inhomogeneity-induced [27] and transverse-geometric timing jitters [38,39], to be 19.4 ps. Based on these calculations, we conclude that the total timing jitter we measured, 20.1 ps, of the X-ray SNSPD was dominated by the intrinsic timing jitter, and we note that this seemingly larger timing jitter, compared with the lowest value, 3.4 ps [40], measured in the visible region, was obtained on an X-ray SNSPD with a practically large area, rather than on a short nanowire [40].

The intrinsic timing jitter fundamentally determines the time-resolving capability of the X-ray SNSPD and in many applications, for example, the above-mentioned celestial GPS by detecting the X-ray from pulsars, it affects the precision of the positioning [47]. However, two important techniques could further enhance the timing and positioning precision if they were applied independently or jointly. The first one is repeated measurements of the arrival times of the X-ray photons—the *N* events of detection of X-ray photons can reduce the uncertainty of the estimation of the most likely arrival time relative to a reference by a factor of $\sqrt{N}$. This fact has been well known in visible and near-IR photon-counting light detection and ranging (LiDAR) to enhance the depth resolution [48]. However, similarly to the photon-counting LiDAR, the cost would be longer time for the measurements. The second is the differential timing measurement [49]—by placing two ‘identical’ X-ray SNSPDs side by side and measuring the difference in the arrival times of the X-ray photons to these two detectors, rather than measuring the individual time difference relative to the reference as we did before. A portion of the extrinsic timing jitter induced by the fluctuations shared by both X-ray SNSPDs, for example, the timing jitter from the reference signal, could possibly be annihilated this way.

We implemented repeated differential timing measurements. Figure 3(a) presents the schematics of the experiment. Two ‘identical’, $50\times 50\, \mu{\rm m}^2$ X-ray SNSPDs were fabricated on the same chip and spaced by 100 *μ*m. The values of their switching current were measured to be 1803 and 1798 *μ*A and they were biased at 1781 and 1761 *μ*A. The X-ray pulses illuminated both detectors; however, because of the low flux of the X-ray photons, we note that the probability that both detectors received X-ray photons from the same pulse was negligibly low. The output voltage signals from these two X-ray SNSPDs were combined electrically by an RF power combiner, and then the combined signal was used as the trigger. The time delays between this combined signal and the reference signal, the output from the fast photodetector, were measured by the oscilloscope. In total, 40 000 time delays of the X-ray photon counts were measured. We used the following algorithm to process the data. We grouped the 40 000 time delays into 50 groups so that each group contained 800 time delays. In each group, for the time delays between 200 and 300 ps, we fitted them with an exponentially modified Gaussian function; for the time delays between 280 and 400 ps, we fitted them with another. Figure 3(b) presents the data and the fittings. There was a 20-ps overlap, meaning that the data from 280 to 300 ps were used in both fittings. We note that this overlap did not affect the results.

The upper panel in Fig. 3(c) presents the timings of the first peaks of the fitted curves, $t{\rm _1^p}$; the solid black line presents the mean value, $\overline{t}{\, \rm _1^p}$, which is 242.51 ps, and the dashed lines present the standard deviation, σ_1_, which is 0.94 ps. Similarly, the middle panel of Fig. 3(c) presents the timings of the second peaks of the fitted curves, $t{\rm _2^p}$; the solid black line presents the mean value, $\overline{t}{\, \rm _2^p}$, which is 319.73 ps, and the dashed lines present the standard deviation, σ_2_, which is 1.21 ps. Considering the 21-ps FWHM timing jitter of each of the two particular X-ray SNSPDs and 800 X-ray photon counts in each group (then, approximately, *N* = 400), σ_1_ and σ_2_ should, theoretically, be compared with $(21/$√${N})/2.35=0.45$ ps. The enhancement of the timing precision, compared with the single-shot timing precision, was contributed from the repeated measurements. The bottom panel in Fig. 3(c) presents the differential timing, $\Delta t= t{\rm _2^p} - t{\rm _1^p}$; the solid black line presents the mean value, $\overline{\Delta t}$, which is 77.23 ps, and the dashed lines present the standard deviation, σ_Δ_, which is 1.24 ps. To compare with σ_1_ and σ_2_, this number needs to be divided by a factor of $\sqrt{2}$, and then we obtained the standard deviation of the differential timing, $(1/\sqrt{2})\sigma _{\Delta }$, which was 0.87 ps and indeed reduced compared with σ_1_ and σ_2_. We note that the reduction in the specific value of the timing precision by the differential timing did not seem significant in our experiment; however, the principle demonstrated here can be equally well applied to applications such as celestial GPS where the cancelation of the external timing jitter could be prominent [49].

## CONCLUSIONS

In conclusion, we have demonstrated a free-running X-ray SNSPD with 20.1-ps timing jitter and with a practically large active area of $50\times 50\, \mu{\rm m}^2$. To our knowledge, this work is the first systematic characterization of the timing properties of SNSPDs in the soft X-ray band (see Table I within the online supplementary material for a list of X-ray SNSPDs published in the literature so far), and it shows that X-ray SNSPDs are ideal detectors of choice for ultrafast X-ray photon counting. The timing resolution and precision have been enhanced by a factor of 4 compared with the best existing X-ray photon counters reported before this work [15]. Furthermore, we demonstrate that repeated differential timing measurements can further enhance the timing precision. The state-of-the-art timing performance of X-ray SNSPDs reported here was enabled by combined engineering optimizations and implementations in nanofabrication and testing, and further optimizations of the film depositions and fabrications may further improve the timing precision of X-ray SNSPDs. We therefore believe that this work significantly advances ultrafast X-ray photonics [11] and that the X-ray SNSPDs demonstrated here may find direct applications in the ground demonstration of X-ray pulsar navigation that China has already launched [8], X-ray astronomy [2], nuclear sciences [4,5] and medical radiography [3].

## METHODS

### Nanofabrication of the X-ray SNSPDs

We fabricated the X-ray SNSPDs presented in this paper at Nanjing University using a nanofabrication process similar to that reported previously [50]. Here, very briefly, 150-nm-thick silicon nitride was deposited on top of a silicon wafer. We sputtered a 100-nm-thick NbN film by reactive sputtering at room temperature. The sheet resistance of the NbN film, measured at room temperature, was $R_{\rm {sq}} =16\, \Omega /\Box$. The critical temperature of the film was measured to be *T*_c_ = 11 K. The sheet inductance at 2.5 K was ≈2.45 pH/$\Box$.

We patterned the devices using 400-nm-thick positive-tone electron-beam resist (AR-P 6200.13) and scanning-electron-beam lithography with an accelerating voltage of 100 kV. The electron-beam lithography contained two steps of exposure. The first step was to pattern the nanowire. To reduce the possible edge roughness of the nanowires, we used a low beam current of 100 pA and a small step size of 2 nm. The second step was to pattern the electrodes, and we used a beam current of 15 nA and a step size of 5 nm. We developed the chips in AR600-546 for 1 min at 20 °C and rinsed them in de-ionized water for another 1 min. To transfer the pattern to the NbN film, we performed CF_4_ reactive-ion etching at 80 W for 3 min with a low pressure of 0.5 Pa to obtain more vertical sidewalls of the nanowires. After etching, we removed the residual AR-P 6200.13 layer in N-methyl pyrrolidone at 80 °C for 5 min and then spin coated a new layer of 400-nm-thick AZ1500 photoresist to protect the device from oxidation and cutting damage. After dicing, AZ1500 was removed with acetone, and we bonded the detectors with gold wires for testing.

### Generation of the femtosecond X-ray pulses

We set up a femtosecond X-ray source, which was excited by an ultrafast laser. The laser source was the Spfire Ace-1001HP laser produced by Spectra-Physics, with its output wavelength centered at 800 nm, peak power of 6 W, pulse width of 0.1 ps (see Fig. 9d within the online supplementary material for the measurement by an autocorrelator) and repetition rate of 1 kHz. The X-ray pulses were steadily generated by illuminating the targets with the laser pulses. The targets made of different materials emitted X-ray pulses with different energy spectra. Specifically, in our home-built setup, the laser deposited its energy onto the surface of a polished 3-inch target in the vacuum chamber through a sapphire window. We used a lens with a focal length of 75 mm to focus the beam of the laser. Both the target and the lens were placed in vacuum chambers with a pressure of 5 × 10^−2^ mbar; between the target and the lens, a thin Mylar film was used to avoid sputtering on the lens while the Mylar film was transparent to the incident light. The target rotated at a speed of 120°/s and moved along the anti-diagonal of the *XY* coordinate plane by 30 *μ*m every 3 s. The movement of the target avoided the laser spot from illuminating the same location; in this way, a target could be used for 2–3 h as the X-ray source without the need for replacement. On the other hand, the movement of the target induced vibration of the surface by $<300\, \mu$m, which further induced timing jitter (Fig. 9e within the online supplementary material). The vacuum chamber containing the target was connected to the pulse-tube cryostat by a metallic corrugated pipe with a length of 160 mm. The X-ray photons illuminated the X-ray SNSPD through a 50-*μ*m-thick, 10-mm-diameter beryllium window mounted on the cryostat, and two polyimide windows mounted on two stages of thermal shields. We employed an electromagnetic shield containing conductive fibres to reduce electromagnetic interference to the readout circuitry and, thus, to reduce noise-induced timing jitter.

### Measurements of timing jitter

The experiment was conducted using a pulse-tube cryocooler at a base temperature of 2.5 K. The signal from the X-ray SNSPD was transmitted through a homemade bias-T to avoid thermal latching at a high bias current. Instead of using a shunt resistor [33], we replaced the constant-current circuit with a constant-voltage circuit that provided a low-impedance path to avoid latching and optimized the signal-to-noise ratio of the output signals. In Figs 1(b) and 2(a), the resistance, inductance and capacitance of *R*_b_, *L*_b_ and *C*_b_ are 5 Ω, 500 nH and 10 pF, respectively. The reduced resistance adapts to the lower hotspot resistance due to the thicker NbN film. Then, the pulse was amplified with a low-noise amplifier LNA-650 at room temperature. The amplifier has a nominal gain of 50 dB, a nominal bandwidth of 600 MHz and a low-frequency cutoff of 30 kHz. We note that the output signals from the X-ray SNSPDs saturated the RF amplifier in our experiments.

The method for measuring the timing jitter of X-ray SNSPDs was similar to that used for measuring the timing jitter of ‘regular’ SNSPDs working in the near-IR range [25,26]. A small fraction of the laser beam was split off and was sent into a fast photodetector (EOT ET-4000), whose output signals served as references for the measurements of time delays and timing jitter. According to the specification of the fast photodetector, the 10% to 90% time constant of the rising edges of the reference voltage signals was measured to be <30 ps; its cutoff frequency was higher than 12.5 GHz. We measured the timing jitter of the fast photodetector by using two such detectors, and the measured timing jitter of each was 0.98 ps (Fig. 9c within the online supplementary material). The reference signals and the output voltage signals from the X-ray SNSPD were sent to a high-speed, real-time oscilloscope (Keysight MSOV334A). Timing jitter was measured at its maximum sampling rate of 80 Gb/s.

## Supplementary Material

nwad102_Supplemental_File

## References

[1] Haase A , Landwehr G, Umbach E et al. Röntgen Centennial: X-Rays in Natural and Life Sciences. Singapore: World Scientific, 1997. 10.1142/3428

[2] Cunningham T , Wheatley PJ, Tremblay PE et al. A white dwarf accreting planetary material determined from X-ray observations. Nature 2022; 602: 219–22. 10.1038/s41586-021-04300-w 35140386

[3] Fan C , Fan M, Orlando BJ et al. X-ray and cryo-EM structures of the mitochondrial calcium uniporter. Nature 2018; 559: 575–9. 10.1038/s41586-018-0330-9 29995856 PMC6368340

[4] Milathianaki D , Boutet S, Williams G et al. Femtosecond visualization of lattice dynamics in shock-compressed matter. Science 2013; 342: 220–3. 10.1126/science.1239566 24115435

[5] Qian G , Wang J, Li H et al. Structural and chemical evolution in layered oxide cathodes of lithium-ion batteries revealed by synchrotron techniques. Natl Sci Rev 2022; 9: nwab146. 10.1093/nsr/nwab146 35145703 PMC8824737

[6] Witze A . NASA test proves pulsars can function as a celestial GPS. Nature 2018; 553: 261–3. 10.1038/d41586-018-00478-8 32094601

[7] Shemar S , Fraser G, Heil L et al. Towards practical autonomous deep-space navigation using X-ray pulsar timing. Exp Astron 2016; 42: 101–38. 10.1007/s10686-016-9496-z

[8] Zhang X , Shuai P, Huang L et al. Mission overview and initial observation results of the X-ray pulsar navigation-I satellite. Int J Aerosp Eng 2017; 2017: 8561830. 10.1155/2017/8561830

[9] Chapman HN , Barty A, Bogan MJ et al. Femtosecond diffractive imaging with a soft-X-ray free-electron laser. Nat Phys 2006; 2: 839–43. 10.1038/nphys461

[10] Öström H , Öberg H, Xin H et al. Probing the transition state region in catalytic CO oxidation on Ru. Science 2015; 347: 978–82. 10.1126/science.1261747 25722407

[11] Sellberg JA , Huang C, McQueen TA et al. Ultrafast X-ray probing of water structure below the homogeneous ice nucleation temperature. Nature 2014; 510: 381–4. 10.1038/nature13266 24943953

[12] Hu J , Cao Z. Water science on the molecular scale: new insights into the characteristics of water. Natl Sci Rev 2014; 1: 179–81. 10.1093/nsr/nwt015

[13] Hartmann N , Hartmann G, Heider R et al. Attosecond time–energy structure of X-ray free-electron laser pulses. Nat Photon 2018; 12: 215–20. 10.1038/s41566-018-0107-6

[14] Xie J , Wagner R, Demarteau M et al. First experimental demonstration of time-resolved X-ray measurements with next-generation fast-timing MCP-PMT. Nucl Instrum Methods Phys Res A 2019; 927: 287–92. 10.1016/j.nima.2019.02.057

[15] Baron AQ , Kishimoto S, Morse J et al. Silicon avalanche photodiodes for direct detection of X-rays. J Synchrotron Radiat 2006; 13: 131–42. 10.1107/S090904950503431X 16495613

[16] Kishimoto S , Toda A. High-energy and high-rate X-ray measurements using HfO_2_ nanoparticle-loaded plastic scintillator. IEEE Trans Nucl Sci 2021; 68: 165–72. 10.1109/TNS.2020.3048943

[17] Okada S , Bennett D, Curceanu C et al. First application of superconducting transition-edge sensor microcalorimeters to hadronic atom X-ray spectroscopy. Prog Theor Exp Phys 2016; 2016: 091D01. 10.1093/ptep/ptw130

[18] Day PK , LeDuc HG, Mazin BA et al. A broadband superconducting detector suitable for use in large arrays. Nature 2003; 425: 817–21. 10.1038/nature02037 14574407

[19] Ohkubo M , Ukibe M, Saito N et al. Effects of spatial nonuniformity of superconducting-tunnel-junction ion detectors on mass spectroscopy. IEEE Trans Appl Supercond 2005; 15: 932–5. 10.1109/TASC.2005.850126

[20] Gol’tsman G , Okunev O, Chulkova G et al. Picosecond superconducting single-photon optical detector. Appl Phys Lett 2001; 79: 705–7. 10.1063/1.1388868

[21] Takesue H , Nam SW, Zhang Q et al. Quantum key distribution over a 40-dB channel loss using superconducting single-photon detectors. Nat Photon 2007; 1: 343–8. 10.1038/nphoton.2007.75

[22] Zhang C , Gao T, Cao Y et al. The facilities and performance of TianQin laser ranging station. Class Quantum Gravity 2022; 39: 125005. 10.1088/1361-6382/ac6d3e

[23] Xia F , Gevers M, Fognini A et al. Short-wave infrared confocal fluorescence imaging of deep mouse brain with a superconducting nanowire single-photon detector. ACS Photon 2021; 8: 2800–10. 10.1021/acsphotonics.1c01018

[24] Li B , Liu Y, Tong S et al. BER analysis of a deep space optical communication system based on SNSPD over double generalized gamma channel. IEEE Photon J 2018; 10: 1–7. 10.1109/JPHOT.2018.2868738

[25] Zhao Q , Zhang L, Jia T et al. Intrinsic timing jitter of superconducting nanowire single-photon detectors. Appl Phys B 2011; 104: 673–8. 10.1007/s00340-011-4574-4

[26] You L , Yang X, He Y et al. Jitter analysis of a superconducting nanowire single photon detector. AIP Adv 2013; 3: 072135. 10.1063/1.4817581

[27] Cheng Y , Gu C, Hu X. Inhomogeneity-induced timing jitter of superconducting nanowire single-photon detectors. Appl Phys Lett 2017; 111: 062604. 10.1063/1.4985226

[28] Kozorezov A , Lambert C, Marsili F et al. Fano fluctuations in superconducting-nanowire single-photon detectors. Phys Rev B 2017; 96: 054507. 10.1103/PhysRevB.96.054507

[29] Allmaras JP , Kozorezov AG, Korzh BA et al. Intrinsic timing jitter and latency in superconducting nanowire single-photon detectors. Phys Rev Appl 2019; 11: 034062. 10.1103/PhysRevApplied.11.034062

[30] Inderbitzin K , Engel A, Schilling A et al. An ultra-fast superconducting Nb nanowire single-photon detector for soft X-rays. Appl Phys Lett 2012; 101: 162601. 10.1063/1.4759046

[31] Inderbitzin K , Engel A, Schilling A. Soft X-ray single-photon detection with superconducting tantalum nitride and niobium nanowires. IEEE Trans Appl Supercond 2013; 23: 2200505. 10.1109/TASC.2012.2234496

[32] Zhang X , Wang Q, Schilling A. Superconducting single X-ray photon detector based on W_0.8_Si_0.2_. AIP Adv 2016; 6: 115104. 10.1063/1.4967278

[33] Yang C , Si M, Zhang X et al. Large-area TaN superconducting microwire single photon detectors for X-ray detection. Opt Express 2021; 29: 21400–8. 10.1364/OE.422581 34265928

[34] Branny A , Didier P, Zichi J et al. X-ray induced secondary particle counting with thin NbTiN nanowire superconducting detector. IEEE Trans Appl Supercond 2021; 31: 1–5. 10.1109/TASC.2021.3066578

[35] Perez de Lara D , Ejrnaes M, Casaburi A et al. Feasibility investigation of NbN nanowires as detector in time-of-flight mass spectrometers for macromolecules of interest in biology (proteins). J Low Temp Phys 2008; 151: 771–6. 10.1007/s10909-008-9745-2

[36] Yang JK , Kerman AJ, Dauler EA et al. Modeling the electrical and thermal response of superconducting nanowire single-photon detectors. IEEE Trans Appl Supercond 2007; 17: 581–5. 10.1109/TASC.2007.898660

[37] Calandri N , Zhao QY, Zhu D et al. Superconducting nanowire detector jitter limited by detector geometry. Appl Phys Lett 2016; 109: 152601. 10.1063/1.4963158

[38] Wu H , Gu C, Cheng Y et al. Vortex-crossing-induced timing jitter of superconducting nanowire single-photon detectors. Appl Phys Lett 2017; 111: 062603. 10.1063/1.4997930

[39] Vodolazov DY . Minimal timing jitter in superconducting nanowire single-photon detectors. Phys Rev Appl 2019; 11: 014016. 10.1103/PhysRevApplied.11.014016

[40] Korzh B , Zhao QY, Allmaras JP et al. Demonstration of sub-3 ps temporal resolution with a superconducting nanowire single-photon detector. Nat Photon 2020; 14: 250–5. 10.1038/s41566-020-0589-x

[41] Marsili F , Verma VB, Stern JA et al. Detecting single infrared photons with 93% system efficiency. Nat Photon 2013; 7: 210–4. 10.1038/nphoton.2013.13

[42] Esmaeil Zadeh I , Los JW, Gourgues RB et al. Efficient single-photon detection with 7.7 ps time resolution for photon-correlation measurements. ACS Photon 2020; 7: 1780–7. 10.1021/acsphotonics.0c00433

[43] Clem JR , Berggren KK. Geometry-dependent critical currents in superconducting nanocircuits. Phys Rev B 2011; 84: 174510. 10.1103/PhysRevB.84.174510

[44] Corde S , Phuoc KT, Lambert G et al. Femtosecond X-rays from laser-plasma accelerators. Rev Mod Phys 2013; 85: 1. 10.1103/RevModPhys.85.1

[45] Berger MJ , Hubbell J, Seltzer S et al. XCOM: Photon Cross Sections Database Share. https://www.nist.gov/pml/xcom-photon-cross-sections-database (Retrieved 16 September 2022).

[46] Sidorova M , Semenov A, Hübers HW et al. Physical mechanisms of timing jitter in photon detection by current-carrying superconducting nanowires. Phys Rev B 2017; 96: 184504. 10.1103/PhysRevB.96.184504

[47] Wang Y , Zheng W, Sun S et al. X-ray pulsar-based navigation using time-differenced measurement. Aerosp Sci Technol 2014; 36: 27–35. 10.1016/j.ast.2014.03.007

[48] Massa JS , Buller GS, Walker AC et al. Time-of-flight optical ranging system based on time-correlated single-photon counting. Appl Opt 1998; 37: 7298–304. 10.1364/AO.37.007298 18301562

[49] Guo F , Zhang X, Wang J. Timing group delay and differential code bias corrections for BeiDou positioning. J Geod 2015; 89: 427–45. 10.1007/s00190-015-0788-2

[50] Guo S , Chen Q, Pan D et al. Fabrication of superconducting niobium nitride nanowire with high aspect ratio for X-ray photon detection. Sci Rep 2020; 10: 9057. 10.1038/s41598-020-65901-5 32494024 PMC7271163

