# Supplementary material for: High-timing-precision detection of single X-ray photons by superconducting nanowires
Source: Natl Sci Rev. 2023 Apr 18;11(1):nwad102. doi: 10.1093/nsr/nwad102 (PMC10727846; doi:10.1093/nsr/nwad102)
Supplement: nwad102_Supplemental_File [file nwad102_supplemental_file.pdf]

National Science Review

Supplementary information:

High-timing-precision detection of single X-ray photons by superconducting nanowires

Shuya Guo,[1,†] Jingrou Tan,[1,†] Hengbin Zhang,[2,†] Jinguang Wang,[3,†] Tianhao Ji,[1,†] Labao Zhang,[1,4,*] Xiaolong Hu,[5,6,*] Jian Chen,[1,*] Jun Xie,[2,*] Kai Zou,[5,6] Yun Meng,[5,6] Xiaomin Bei,[2] Ling-An Wu,[3] Qi Chen,[1] Hao Wang,[1] Xuecou Tu,[1] Xiaoqing Jia,[1,4] Qing-Yuan Zhao,[1] Lin Kang,[1,4] and Peiheng Wu[1,4,*]

[1]*Research Institute of Superconductor Electronics, Nanjing University, Nanjing 210093, China*
[2]*Qian Xuesen Laboratory of Space Technology, Beijing, 100094, China*
[3]*Beijing National Laboratory of Condensed Matter Physics, Institute of Physics, Beijing 100190, China*
[4]*Hefei National Laboratory, Hefei 230088, China*
[5]*School of Precision Instrument and Optoelectronic Engineering, Tianjin University, Tianjin 300072, China*
[6]*Key Laboratory of Optoelectronic Information Science and Technology, Ministry of Education, Tianjin 300072, China*

Contents of this file:

Supplementary Figures 1, 2, 3, 4, 5, 6, 7, 8, 9, 10

Supplementary Notes 1, 2, 3

Supplementary Tables I

Supplementary References

[†] These authors contributed equally to this work.

[*] Lzhang@nju.edu.cn
[*] xiaolonghu@tju.edu.cn
[*] chenj63@nju.edu.cn
[*] xiejuncast001@163.com
[*] phwu@nju.edu.cn

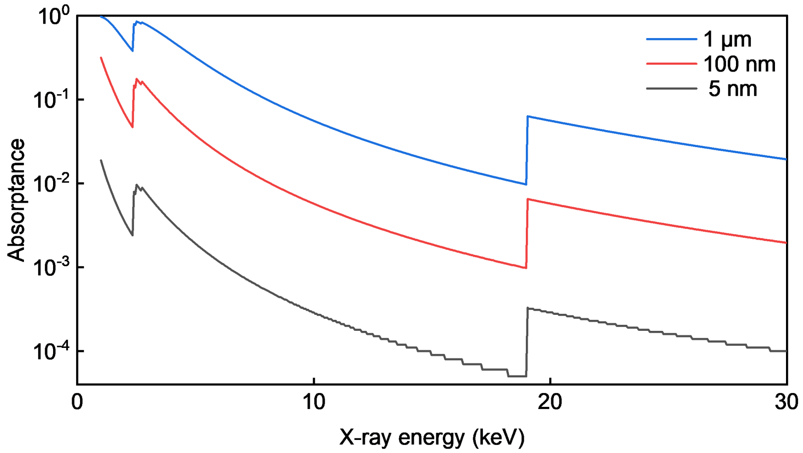

Supplementary Figure 1. Simulated absorptance spectra of the NbN films as a function of X-ray energy. Three thicknesses, 5 nm, 100 nm, and 1 μm, of the NbN films were simulated. The attenuation coefficients were obtained from ref[S1].

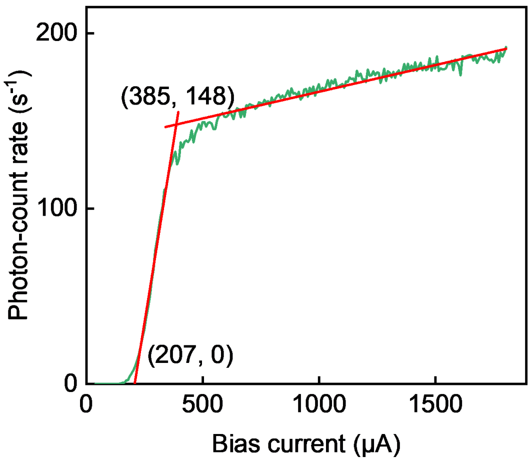

Supplementary Figure 2. The photon-count rate vs. bias current. The red lines are two-segment fittings by two linear functions.

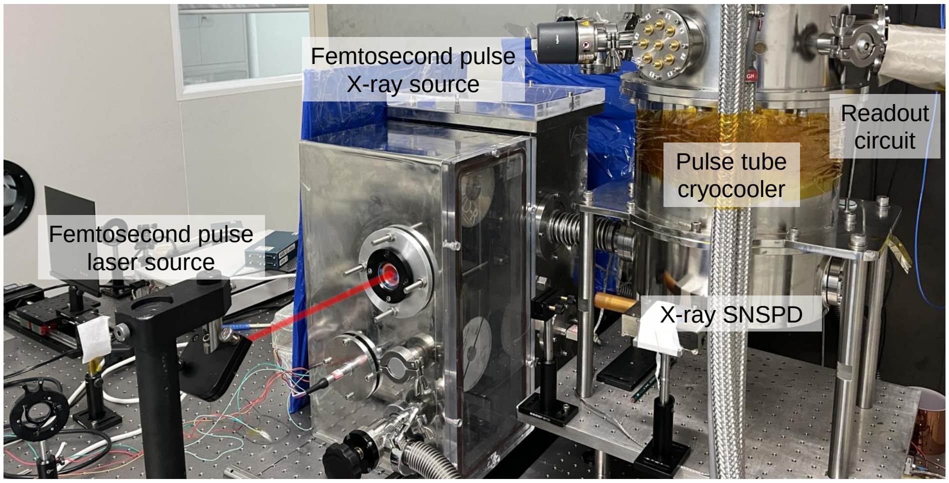

Supplementary Figure 3. A photograph of the experimental setup with its major components labeled. The red beam was artificially added on the photo, showing the femtosecond laser beam.

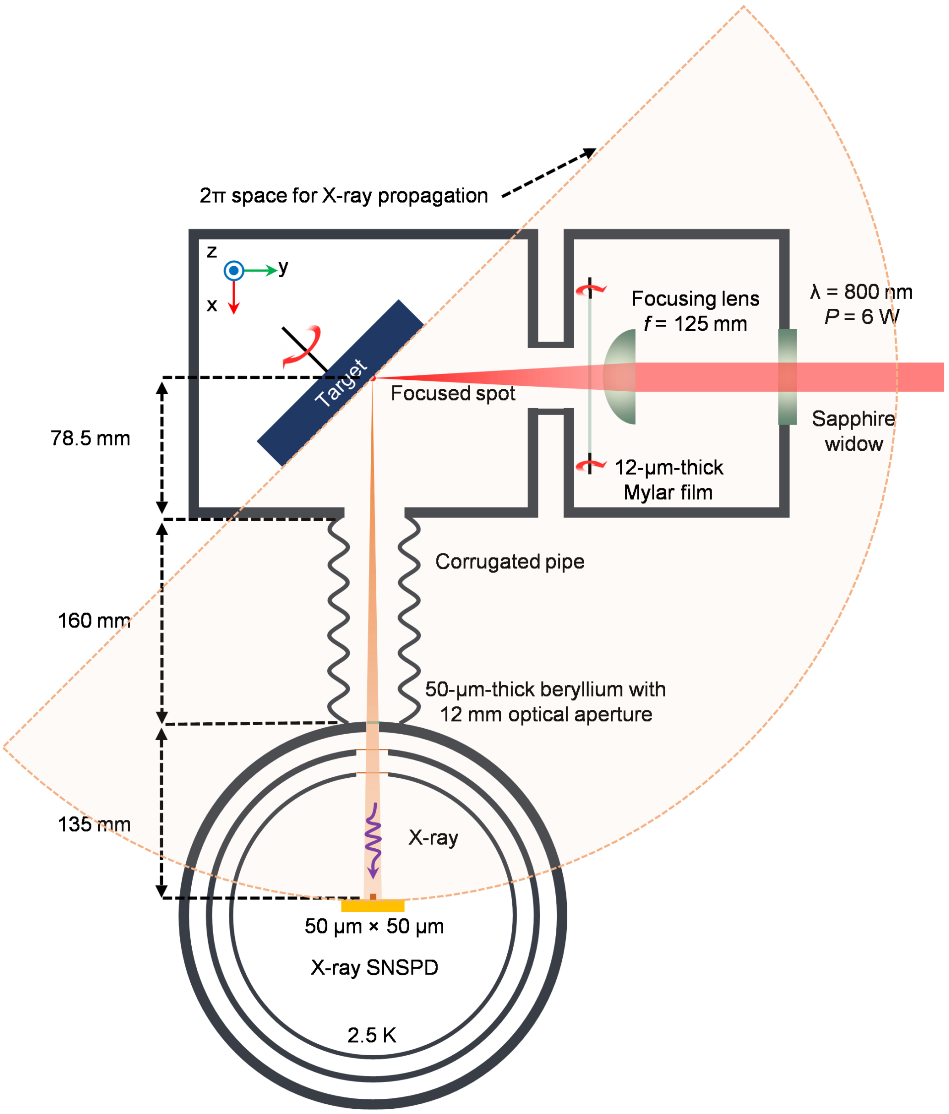

Supplementary Figure 4. A schematic diagram of the experimental setup with dimensions labeled.

Supplementary Note 1. Estimation of the flux of the X-ray photons

We estimated the average flux of the X-ray photons received by the X-ray SNSPD. The discussions here are for the titanium target. The total number of the K_α X-ray photons in the 2π hemisphere excited by one laser pulse was 5×10^7 photons/shot. The number of the X-ray photons generated by bremsstrahlung (the broad band X-ray) can be estimated by the ratio of the total area under the energy spectrum to the area under the K_α peak. The ratio was 3.6. So the total number of the X-ray photons per shot was $n = 1.8 \times 10^8$ photons/shot. We assume that the emission of the X-ray photons was a uniform distribution over the hemisphere. The surface area of the hemisphere was $S_1 = 2\pi r^2 = 8.76 \times 10^5$ mm^2, where $r = 78.5 + 160 + 135 = 373.5$ mm. The area of the X-ray SNSPD was $S_2 = 0.5 \times 50$ μm $\times$ 50 μm . The factor, 0.5, was the fill factor. So the average number of the X-ray photons received by the X-ray SNSPD per shot was $\overline{n} = n \times \frac{S_2}{S_1} = 0.26$ photons/shot, conforming $\overline{n} << 1$ photons/shot. Assuming a Poissonian statistics of the X-ray photon numbers in the pulses, we calculated the probability that a pulse contains n photons, $P(n)$, to be $P(0) = 0.77$, $P(1) = 0.2$, $P(2) = 0.026, ...$ Therefore, $P(n \geq 2) = 0.03 << 1$.

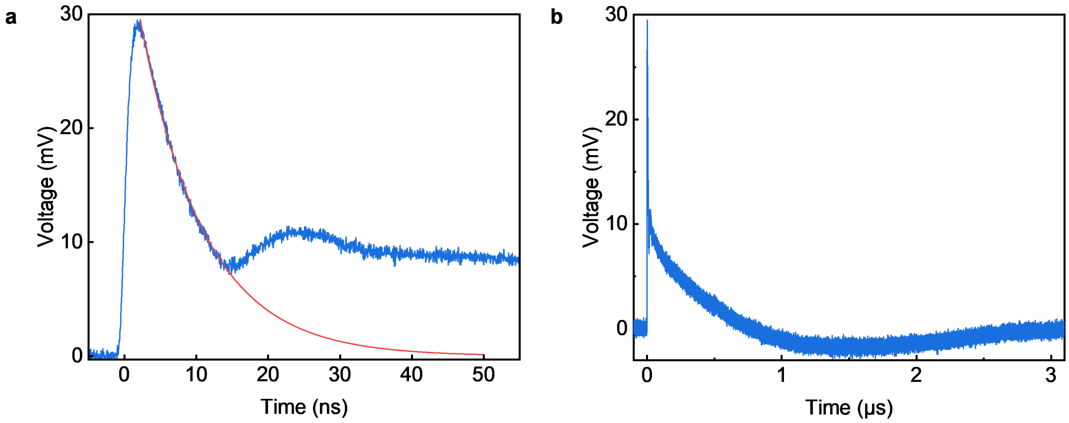

Supplementary Figure 5. (a) Oscilloscope waveform of an output pulse from the X-ray SNSPD without an amplifier, while the solid red line shows the fitting curve of the exponential-decay function. (b) The waveform in a longer time scale.

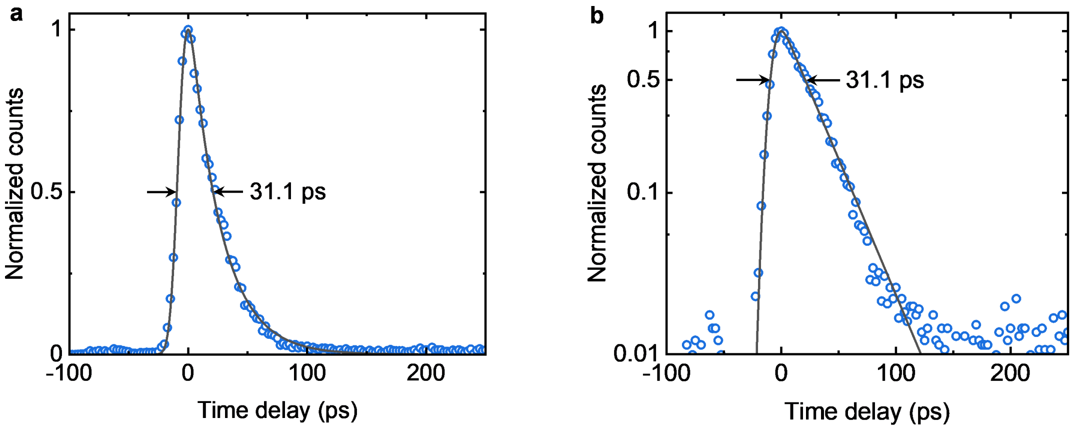

Supplementary Figure 6. Typical normalized histograms of the time delays presented in the (a) linear and (b) semi-log plots. The blue open circles are the measured data and the gray lines are the fitted curves by the exponentially-modified Gaussian functions [S2]. The full width at the half maxima of each fitting curve was used as the value of timing jitter.

Supplementary Note 2. Fitting of the histogram of the time delay

We fitted each histogram of the time delays with an exponentially-modified Gaussian function [S2],

$$H(t) = \frac{A}{2\tau_0}\exp\{\frac{1}{2\tau_0}[\frac{\sigma^2}{\tau_0} - 2(t - t^{\mathrm{p}})]\}\{1 - \mathrm{erf}[\frac{\frac{\sigma^2}{\tau_0} - (t - t^{\mathrm{p}})}{\sqrt{2}\sigma}]\}, \tag{S1}$$

where erf(·) is the error function, A, σ, τ_0, and t^{p} are the fitting parameters. We used the full width at half maxima (FWHM) of the $H(t)$ to quality the timing jitter.

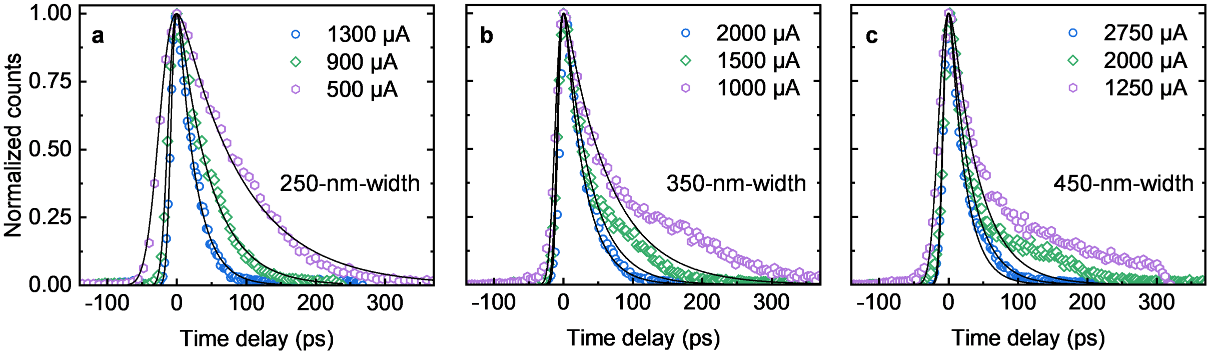

Supplementary Figure 7. Normalized histograms of the time delays of the X-ray SNSPDs with different widths. (a) 250-nm-wide nanowire; (b) 350-nm-wide nanowire; (c) 450-nm-wide nanowire.

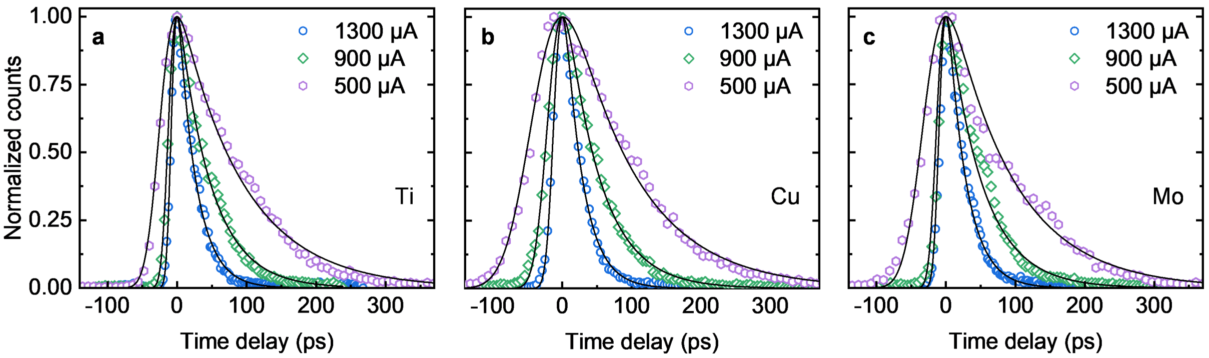

Supplementary Figure 8. Normalized histograms of time delays measured by an X-ray SNSPD (50 µm × 50 µm area and 250-nm-width nanowire) and with three difference X-ray sources. Data for the Ti (a), Cu (b) and Mo (c) targets are shown for comparison.

Supplementary Note 3. Dependence of timing jitter on the geometries of the X-ray SNSPDs

When characterizing the X-ray SNSPDs with different widths of nanowires, we noticed that the tails of the time-delay histograms showed different shapes. Supplementary Fig. 7 presents three widths (250 nm, 350 nm, and 450 nm) at different bias current. At the bias current below $0.6I_{\mathrm{sw}}$, there was a significant deviation of the histograms from the exponentially-modified Gaussian functions for the 350-nm-wide and 450-nm-wide nanowires. The origin of this deviation might be attributed to the so-called “transverse geometric” timing jitter [S3, S4], which became more significant for wider nanowires at low bias current.

As presented in Supplementary Fig. 8, regardless of the targets generating the X-ray photons, i.e., the energies of the X-ray photons, all the histograms can be well fitted by the exponentially-modified Gaussian functions for the X-ray SNSPD with a 250-nm-wide nanowire.

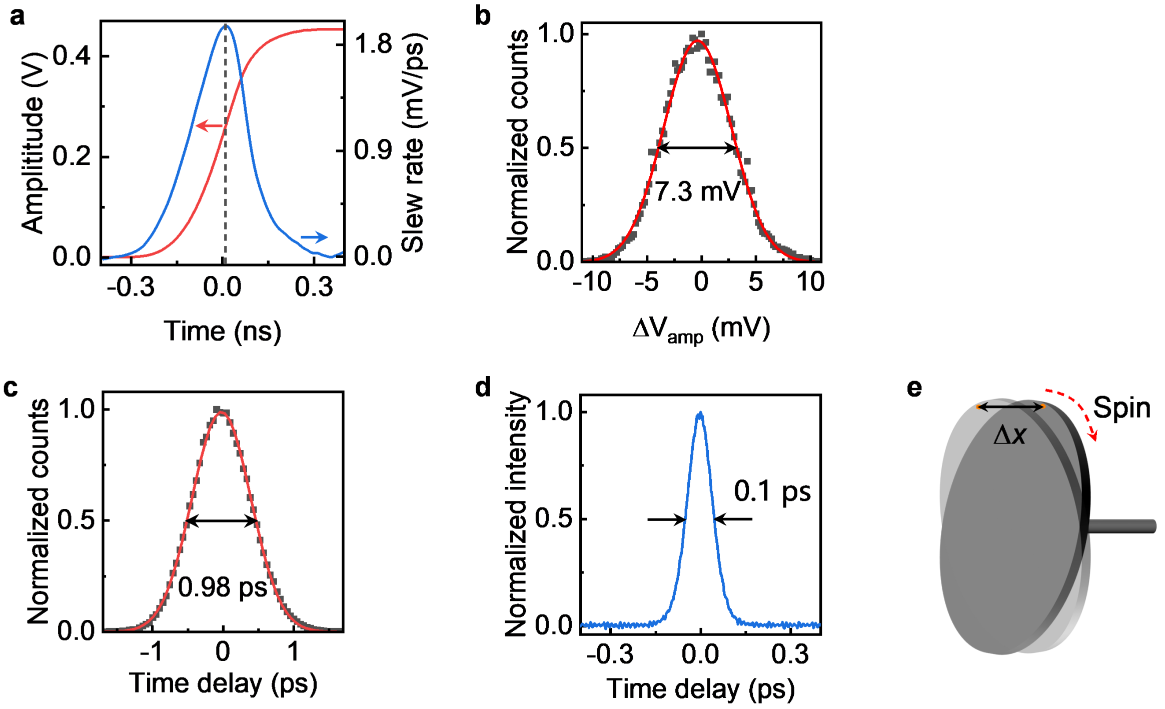

Supplementary Figure 9. **Extrinsic time jitter induced by the measurement system.** (a) The measured average rising edge of the temporal trace of the output voltage pulses from the X-ray SNSPD (red line) and the corresponding slew rate (blue line). The detector was biased at I_b = 1760 µA. The trigger level was set at the point with maximum slew rate (1.96 mV/ps) for the measurements of timing jitter. (b) The histogram of the electronic noise measured by the oscilloscope, showing a FWHM amplitude jitter of 7.3 mV, which contributes 3.73 ps of timing jitter. (c) The histogram of the time delay of the fast photodiode measured with the femtosecond laser, showing timing jitter of 0.98 ps. (d) The pulse width of the femtosecond laser was measured to be 0.1 ps by an autocorrelator. (e) The schematic diagram showing how the vibration of the target resulted in different optical paths and induced timing jitter. The maximum difference of optical paths was approximately 300 µm.

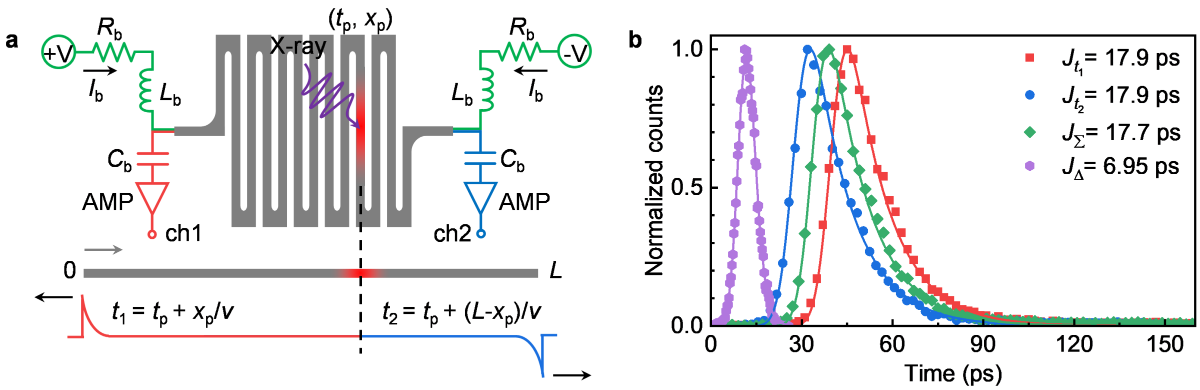

Supplementary Figure 10. **Geometric timing jitter**. (a) Schematic of the dual-port bias and readout circuitry. (b) Normalized histograms of the time delays of $t_1(J_{t_1})$, $t_2(J_{t_2})$, the sum of t_1 and t_2, $[(t_1 + t_2)/2]$, J_Σ, and the difference of $(t_1 - t_2)$, J_Δ, of a 50 µm × 50 µm device with dual-port bias and readout circuitry. This particular device was biased at 1880 µA for these measurements. The geometric timing jitter [S5], $J_\Delta/2$, was calculated to be 3.5 ps.

TABLE I. Comparison of performances of the X-ray SNSPDs published in literature.

	References	Materials	Active area (μm × μm)	Width (nm)	Thickness (nm)	X-ray photon energy (keV)	Latching current	Timing jitter (ps)
Thick film	**This work**	**NbN**	**50 × 50**	**250**	**100**	**1-18**	**without latching**	**20.1**
	Yang et al., 2021 [S6]	TaN	2250 × 2250	2300	100	5.4-10	0.7 I_{sw} @4.6 K	NA
	Zhang et al., 2016 [S7]	$W_{0.8}Si_{0.2}$	41.6 × 28	920	100	5.30, 49.9	0.1 I_{sw} @1.8 K	NA
	Inderbitzin et al., 2013 [S8]	TaN	35 × 33	250	100	5.5-49.9	0.52 I_{sw} @1.84 K	NA
	Inderbitzin et al., 2012 [S9]	Nb	131 × 33	250	100	5.5-49.9	0.055 I_{sw} @1.75 K	NA
Thin film	Branny et al., 2021 [S10]	NbTiN	$\pi \times 5 \times 5$	70	10	4-25	without latching	NA
	Perez et al., 2008 [S11]	NbN	5 × 5	120	5	5.89	without latching	NA

[S1] M. J. Berger, J. Hubbell, S. M. Seltzer, J. Chang, J. S. Coursey, R. Sukumar, D. S. Zucker, and K. Olsen, XCOM: Photon Cross Sections Database Share, website (2013), https://www.nist.gov/pml/xcom-photon-cross-sections-database.

[S2] M. Sidorova, A. Semenov, H.-W. Hübers, I. Charaev, A. Kuzmin, S. Doerner, and M. Siegel, Physical mechanisms of timing jitter in photon detection by current-carrying superconducting nanowires, Physical Review B **96**, 184504 (2017).

[S3] H. Wu, C. Gu, Y. Cheng, and X. Hu, Vortex-crossing-induced timing jitter of superconducting nanowire single-photon detectors, Applied Physics Letters **111**, 062603 (2017).

[S4] D. Y. Vodolazov, Minimal timing jitter in superconducting nanowire single-photon detectors, Physical Review Applied **11**, 014016 (2019).

[S5] N. Calandri, Q.-Y. Zhao, D. Zhu, A. Dane, and K. K. Berggren, Superconducting nanowire detector jitter limited by detector geometry, Applied Physics Letters **109**, 152601 (2016).

[S6] C. Yang, M. Si, X. Zhang, A. Yu, J. Huang, Y. Pan, H. Li, L. Li, Z. Wang, S. Zhang, J. Xia, Z. Liu, H. Guo, and L. You, Large-area TaN superconducting microwire single photon detectors for X-ray detection, Optics Express **29**, 21400 (2021).

[S7] X. Zhang, Q. Wang, and A. Schilling, Superconducting single X-ray photon detector based on $W_{0.8}Si_{0.2}$, AIP Advances **6**, 115104 (2016).

[S8] K. Inderbitzin, A. Engel, and A. Schilling, Soft X-Ray Single-Photon Detection With Superconducting Tantalum Nitride and Niobium Nanowires, IEEE Transactions on Applied Superconductivity **23**, 2200505 (2013).

[S9] K. Inderbitzin, A. Engel, A. Schilling, K. Il'in, and M. Siegel, An ultra-fast superconducting Nb nanowire single-photon detector for soft x-rays, Applied Physics Letters **101**, 162601 (2012).

[S10] A. Branny, P. Didier, J. Zichi, I. E. Zadeh, S. Steinhauer, V. Zwiller, and U. Vogt, X-ray induced secondary particle counting with thin NbTiN nanowire superconducting detector, IEEE Transactions on Applied Superconductivity **31**, 1 (2021).

[S11] D. Perez de Lara, M. Ejrnaes, A. Casaburi, M. Lisitskiy, R. Cristiano, S. Pagano, A. Gaggero, R. Leoni, G. Golt'sman, and B. Voronov, Feasibility investigation of NbN nanowires as detector in time-of-flight mass spectrometers for macromolecules of interest in biology (proteins), Journal of Low Temperature Physics **151**, 771 (2008).
